# Barriers and Facilitators to the Implementation of the Pathways to Comorbidity Care (PCC) Training Package for the Management of Comorbid Mental Disorders in Drug and Alcohol Settings

**DOI:** 10.3389/frhs.2021.785391

**Published:** 2021-11-24

**Authors:** Eva Louie, Vicki Giannopoulos, Andrew Baillie, Gabriela Uribe, Katie Wood, Maree Teesson, Steven Childs, David Rogers, Paul S. Haber, Kirsten C. Morley

**Affiliations:** ^1^Sydney Medical School, Faculty of Medicine and Health, The University of Sydney, Sydney, NSW, Australia; ^2^Drug Health Services, Royal Prince Alfred Hospital, Camperdown, NSW, Australia; ^3^School of Health Sciences, Faculty of Medicine and Health, The University of Sydney, Sydney, NSW, Australia; ^4^The Matilda Centre for Research in Mental Health and Substance Use, The University of Sydney, Sydney, NSW, Australia; ^5^Central Coast Local Health District, Drug and Alcohol Clinical Services, Gosford, NSW, Australia; ^6^Drug and Alcohol Services, Mid North Coast Local Health District, Port Macquarie, NSW, Australia

**Keywords:** barriers, facilitators, implementation, training, comorbidity

## Abstract

**Background:** We have previously reported that the Pathways to Comorbidity Care (PCC) training program for alcohol and other drug (AOD) clinicians improved identification of comorbidity, self-efficacy, and attitudes toward screening and monitoring of comorbidity. We aimed to identify barriers and facilitators of implementation of the PCC training program in drug and alcohol settings.

**Methods:** The PCC training program was implemented across 6 matched sites in Australia as per (1), and 20 clinicians received training. PCC training included seminar presentations, workshops conducted by local “clinical champions,” individual clinical supervision, and access to an online information portal. We examined barriers and facilitators of implementation according to the Consolidated Framework for Implementation Research.

**Results:** Barriers included inner setting (e.g., allocated time for learning) and characteristics of individuals (e.g., resistance). Facilitators included intervention characteristics (e.g., credible sources), inner setting (e.g., leadership), and outer setting domains (e.g., patient needs). Clinical champions were identified as an important component of the implementation process.

**Conclusions:** Barriers included limited specific allocated time for learning. A credible clinical supervisor, strong leadership engagement and an active clinical champion were found to be facilitators of the PCC training program.

## Introduction

Drug and alcohol clinicians are highly likely to encounter patients with comorbid mental health disorders, which may occur in up to 9 out of 10 patients (2). This poses several challenges for drug and alcohol clinicians, who must manage a more complex range of symptoms in a population that are often more reliant on treatment services (3, 4), and who may have to negotiate limited networks between drug and alcohol and mental health services (5). When these problems are not resolved, patients are at risk of not receiving effective treatments and have poorer outcomes (3, 4).

One approach to addressing this gap in service provision is to train clinicians in an evidence-based approach to treating comorbidity that also takes account of the systemic barriers to effective treatment. The integrated care approach is one such model, which promotes the identification, assessment and treatment of both the alcohol and other drug (AOD) use and the mental health disorder within one service (6). Providing care within the one service addresses problems that may be associated with non-cohesive, parallel treatment plans whereby care is delivered by two separate services. Indeed, integrated care has been recommended for use in the Australian clinical guidelines for comorbid mental disorders and substance use (6).

Although research into the effectiveness of interventions designed to improve the uptake of evidence-based treatment models like integrated care has been limited, there is some evidence to suggest that certain training efforts have been effective. For example, one study evaluating the effectiveness of a 2-day workshop and supervision in screening and brief intervention found improvements in the identification, case formulation, and treatment of comorbid mental health disorders by AOD clinicians (7). Another study evaluating the effectiveness of a brief comorbidity training program and clinical supervision for case managers of community mental health teams found improvements in self-efficacy (8). While these studies demonstrated the effectiveness of their respective training programs, they did not explicitly evaluate the effects of the implementation efforts themselves. This is an important distinction, since findings from the field of implementation science consistently demonstrate that the implementation process itself also has implications for implementation outcomes (9, 10). A recent systematic review of evidence-based practise implementation in AOD settings found that only two of the twenty included studies employed a comprehensive implementation framework, and no included studies focused on treating comorbidity (11).

Given the broad array of clinical training and experience, health service leadership, organisational dynamics, and health systems in public health treatment settings, successfully implementing integrated care is a complex undertaking (12). Indeed, bridging the gap between evidence and practise requires systematic assessment of the implementation barriers that exist at multiple levels of healthcare delivery including the patient level, the provider level and the organisational level (13). Within the AOD treatment setting, integrated care might be particularly challenging to implement given the segregated nature of AOD and mental health services. These disrupted networks negatively impact the implementation of integrated care and patients are more likely to “fall through the gaps” or get passed between services (5). The application of a comprehensive implementation framework would help illustrate the challenges specific to the treatment of comorbidity in AOD settings.

A more thorough attempt at designing and evaluating the efficacy and implementation effectiveness of a comorbidity training program for drug and alcohol clinicians was the multi-modal Pathways to Comorbidity Care (PCC) training package. The PCC package was developed to target potential barriers to delivering integrated care for comorbid substance use and mental disorders in AOD settings. These included improving knowledge, attitudes and confidence of AOD clinicians to manage these problems see (14). The multiple modalities of the PCC training were designed to present didactic material to establish a standard of knowledge (resources, seminars) followed by provision of interactive learning (clinical supervision and clinical champions) to problem solve implementation in these settings. The multi-modal design was influenced by previous findings indicating that in AOD settings, multi-level strategies rather than single level strategies, such as those that focus only on the provider, are preferable to facilitate integrated care (15).

We have previously reported that the PCC training package effectively improved the rate of comorbidity identification, increased clinician self-efficacy for managing comorbidity, and improved attitudes toward screening and assessment of comorbidity (1). In addition to these findings, the PPC project involved the systematic evaluation of the barriers and facilitators of the implementation through the application of the Consolidated Framework for Implementation Research (CFIR) (10). The CFIR has been suggested to be suitable model to guide systematic evaluation of multi-level implementation contexts (16). It includes five domains of influence derived from a consolidation of the plethora of terms and concepts generated by implementation researchers: (1) intervention characteristics (e.g., evidence strength and quality, adaptability), (2) outer setting (e.g., patient needs and resources, external policies and incentives), (3) inner setting (e.g., implementation climate, readiness for implementation), (4) individuals involved (e.g., self-efficacy, knowledge, and beliefs about the intervention), and (5) the implementation process (e.g., engaging members of the organisation, executing the innovation). No previous studies have systematically evaluated barriers and facilitators of implementation of comorbidity training according to a validated framework, which is key to refining ongoing training programs and future roll out efforts (11).

This study aimed to report barriers and facilitators of the PCC program using the Consolidated Framework for Implementation Research. The Consolidated Framework for Implementation Research was employed as a guiding framework for determining the specificities of the implementation context, evaluating the implementation and providing a means of assessing the outcome of the implementation.

## Methods

### Study Design

Study methods have been previously described elsewhere (1). Briefly, this was a controlled, before-and-after study (0–9 months) comparing PCC-training vs. control. Three PCC and three control sites were matched according to geographical location across six government AOD outpatient and community health services in NSW, Australia (June 2017–2018). Ethical approval for the study was obtained from the Human Ethics Review Committees of the Sydney Local Health District, South Western Sydney Local Health District, Central Coast Local Health District, Hunter New England Research Ethics and Governance Office which covered two participating services, and Mid North Coast Local Health District (X16-0440 & HREC/16/RPAH/624).

### Participants and Procedures

A signed buy-in from the managers of each site was obtained including a statement that the organisation has endorsed the use of integrated comorbidity management including support for time and resources for clinicians to participate. Potential clinical champions were identified by managers at each PCC site. All participants provided informed consent before taking part in the study. Approximately 12 months after baseline, semi-structured interviews were conducted with clinicians at PCC sites.

### Pathways to Comorbidity Care Intervention

Participants at PCC sites commenced the training program after completing the baseline assessment. The PCC training program has been described in detail previously (14).

Phase 1 (Months 1–3): This was a 12-week non-intensive period of training whereby participants were given access to the online training portal containing various comorbidity resources, the National Comorbidity Guidelines (6) and manuals. Within the following month, a 1-day face to face seminar was conducted at each of the PCC sites (including webinars about comorbid Substance Use and Depression, Anxiety, Trauma, Psychosis, and Bipolar Disorder).

Phase 2 (Months 3–6): This was a 12-week intensive period in which local clinical champions conducted regular group workshops and clinicians received telephone supervision from an experienced senior clinical psychologist (17).

Phase 3 (Months 6–9): Participants were provided prompts to revisit the training portal www.pccportal.org.au. Webinars from Phase 1 were also made available on the portal as booster sessions.

### Data Collection

Between July and September 2018 interviews were conducted with 20 clinicians who participated in the PCC training program. All clinicians providing counselling to patients across 3 public health outpatient drug and alcohol services, distributed throughout the state of New South Wales (inner-metro, outer-metro and regional; with matched controls), were invited to take part in the study. None of the participating clinicians declined to be interviewed. Interviews took place over the phone and were audiotaped and transcribed (KW, GU, EL).

### Outcome Measures

The semi-structured interviews were evaluated according to the CFIR. The CFIR consolidates the concepts generated by implementation research into five domains of influence: (1) intervention characteristics, (2) outer setting, (3) inner setting, (4) individuals involved, and (5) the implementation process (18). Barriers and facilitator outcomes: intervention characteristics, outer setting, inner setting, characteristics of individuals and the implementation process as per the CFIR (see below).

### Coding

Interviews transcriptions were coded (KW, GU, EL) using thematic analysis to identify perspectives and themes, with the CFIR providing a guiding framework for interpretation. Initial themes were identified by EL and KW. Where differences existed in analysis, themes were discussed between the two researchers until consensus was reached. All initial themes and perspectives mapped onto the CFIR constructs.

We developed one codebook before coding the data. In the codebook, we initially included all 39 CFIR constructs and their definitions as codes to capture contextual factors that might influence the implementation of PCC components. These CFIR codes were analytical in that they required the coder to interpret the data and then apply the CFIR code that reflected a potential barrier or facilitator being described. The identification of barriers and facilitators was the main theoretical driver of our study. We applied the CFIR codes to fit the context of the PCC training program by first creating a set of structured and semi-structured interview questions that related directly to the PCC intervention and then identifying which of the CFIR domains were addressed. Consequently, certain subdomains of the CFIR were missing (e.g., inner setting characteristics including tension for change and readiness for implementation were not assessed).

Responses were coded by EL using a directed content analysis approach (19) in which responses were placed in the most relevant domain. If a response could be coded into more than one domain, EL allocated the most appropriate domain. The coding of the interviews was checked by other team members (KM, KW, GU).

### Analysis

To analyse the coded data for the barriers and facilitators of the PCC program, we generated code reports using NVIVO software. This software assisted with the process of grouping segments of text directly from transcribed interviews into categories that could be coded. Each transcription was coded according to each combination of PCC component and CFIR construct. Within each report, data was organised by CFIR domain (e.g., intervention characteristics). We then developed analytic summaries for each CFIR construct and determined whether the component exerted either a positive (strength) or negative (weakness) on implementation.

## Results

### Sample Characteristics

Six AOD services including 3 PCC and 3 control sites participated including 35 participants (*N* = 20 PCC, *N* = 15 control). Baseline characteristics have previously been reported (1). The overall mean age in the PCC group was 51.53 (SD ± 8.14) years, and 75% were female. The majority of participants (60%) had completed a university degree (with no additional post-graduate qualifications), the most common professional role was psychologist (45%) and approximately half of the participants (55%) had done some form of training in mental health during the past 12 months.

### Barriers and Facilitators According to Consolidated Framework for Implementation Research (CFIR)

#### Relevant Theoretical Domains

**Figure 1:**
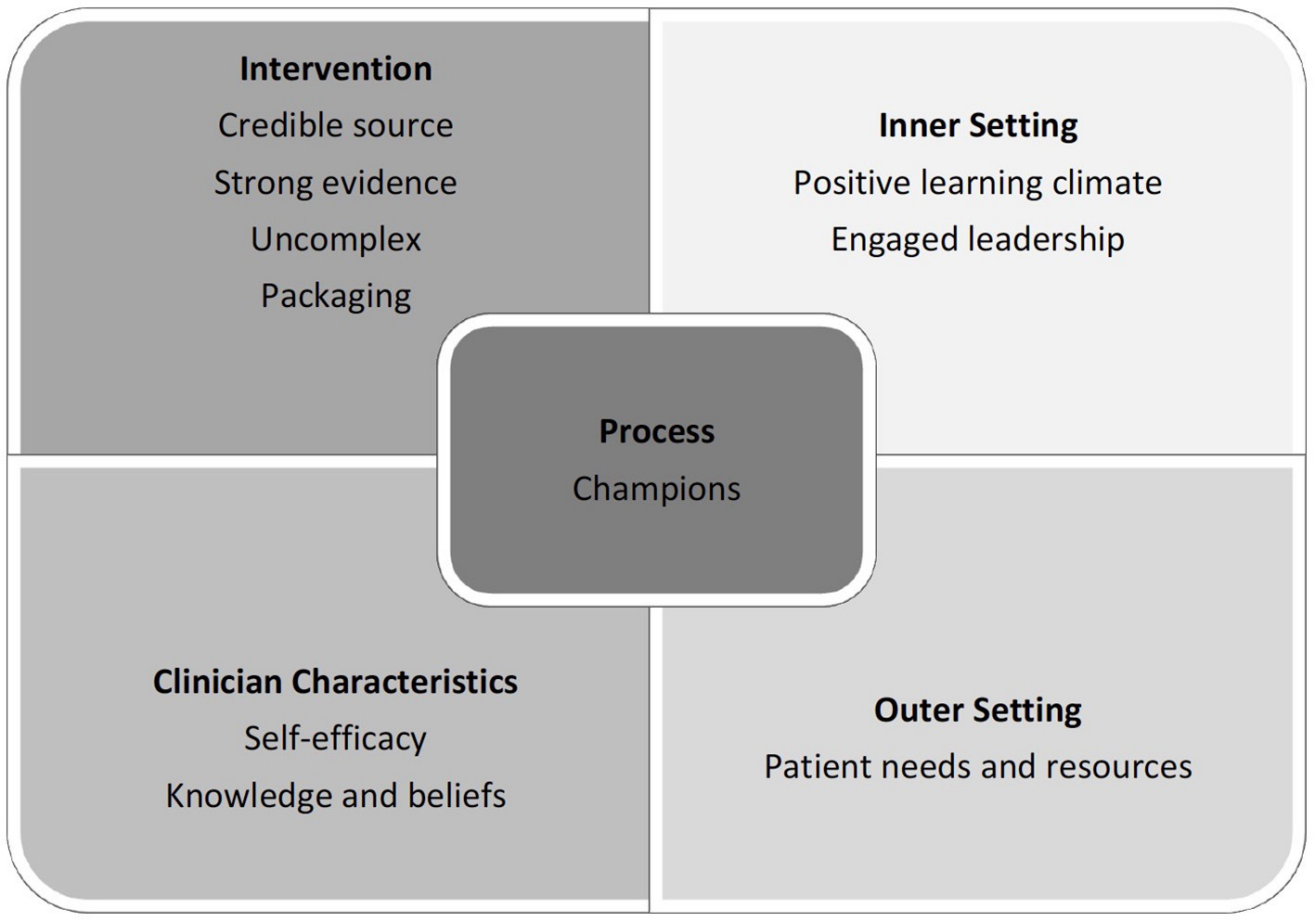
Facilitators of the PCC program as per the domains of the Consolidated Framework for Implementation Research (CFIR).

All barriers and facilitators could be identified within the CFIR (18). Of the 39 CFIR subdomains, 27 were important in understanding the barriers and facilitators to implementing the PCC. Table 1 lists the CFIR domains and corresponding PCC components and whether that domain and component was an implementation weakness or strength. These are briefly outlined below, and the facilitators are summarised in Figure 1. Agreement between ratings of the CFIR subdomains was 100%, and disagreement was 0%, respectively.

**Table 1:** Ratings assigned to CFIR construct.

**I. Intervention characteristics**	
Intervention source	+1
Evidence strength and quality	+1
Relative advantage	0
Adaptability	0
Trialability	M
Complexity (reverse rated)	+1
Design quality and packaging	+1
Cost	M
**II. Outer setting**	
Patient needs and resources	+1
Cosmopolitanism	0
Peer pressure	0
External policy and incentives	M
**III. Inner setting**	
Structural characteristics	M
Networks and communications	0
Culture	M
Implementation climate	0
Tension for change	M
Compatibility	M
Relative priority	M
Organisational incentives and rewards	−1
Goals and feedback	0
Learning climate	+1
Readiness for implementation	M
Leadership engagement	+1
Available resources	0
Access to knowledge and information	0
**Iv. Characteristics of individuals**	
Knowledge and beliefs about the intervention	+1
Self-efficacy	+2
Individual state of change	0
Individual identification with organisation	M
Other personal attributes	−1
**V. Process**	
Planning	0
Engaging	0
Opinion leaders	0
Formally appointed internal implementation leaders	M
Champions	+1
External change agents	M
Executing	0
Reflecting and evaluating	0

*The criteria for rating the constructs reflected the degree of negative or positive evaluations of the implementation. A score of −2 or +2 was given when participants described specific examples of how the construct influenced the implementation, a score of −1 or +1 was given when participants made general statements about the construct influenced the implementation, and a score of 0 was given for a neutral statement. M, Missing, CFIR, Consolidated Framework for Implementation Research*.

##### Intervention Characteristics

Intervention characteristics of the PCC program were considered to be a strength for implementation. Specifically, these were viable intervention sources (e.g., a strong belief that the clinical supervisor had the experience necessary to provide support and feedback, and that it was clear from the beginning that the organisation had approved the intervention), quality of the evidence, the intervention was not too complex, design, and packaging of the intervention (e.g., a strong agreement with the evidence base for the integrated care model). When analysed according to each component of the PCC package, clinicians clearly evaluated the workshop and supervision components much more favourably than the website and didactic seminar components.

##### Inner Setting

The components of the inner setting that were a strength for implementation included fostering of a positive learning climate (e.g., the workshops provided a forum in which clinicians could pass on information to one another), along with leadership engagement. Organisational incentives and rewards appeared to have a negative impact on the implementation process.

##### Outer Setting Factors

These factors were a mild strength of the implementation, especially with regards to the consideration given to patient needs and resources.

##### Characteristics of Individuals

The characteristics of the clinicians such as knowledge and beliefs about the intervention, self-efficacy, and “other personal attributes” revealed mixed results. Knowledge and beliefs and self-efficacy were a positive while other personal attributes (e.g., the thought that one is too senior to listen to others' opinions) negative.

##### Implementation Process

Components of the implementation process that were important and effective included the inclusion of clinical champions.

## Discussion

This study aimed to identify barriers and facilitators of the PCC program using the Consolidated Framework for Implementation Research (CFIR). There has been limited research previously that has systematically evaluated facilitators or barriers of the implementation of training programs aimed to improve the management of mental disorders in AOD settings.

The CFIR analysis revealed that the implementation of the PCC package was mainly facilitated by strong *intervention characteristics* (credible source, uncomplicated approach, high quality design, and convincing evidence) and *outer setting* (good consideration of patient needs and resources) factors. The presence of clinical champions also assisted with the *process* of implementation. *Characteristics of the individuals* involved in the training had mixed effects on the implementation, as self-efficacy was a major strength, while specific personal attributes of participants weakened the impact of the implementation. Mixed results were also found regarding the *Inner setting*, with the creation of a positive learning environment and leadership engagement facilitating implementation, while barriers included a lack of appropriate and sufficient incentives (see Figure 1).

When compared across studies of implementation barriers and facilitators of evidence-based interventions in AOD settings, the CFIR constructs identified as important in this study emulate and extend the accumulating evidence of the field. For instance, with regard to *intervention characteristics*, previous research has revealed that clinicians' perceptions of implementation effectiveness (20) or a lack of clarity about the evidence behind the intervention (21) may influence the uptake of the intervention, and that complex guidelines can be inhibitive (22). This study extends these findings by suggesting that having a credible source of information is just as important as having convincing evidence for it, and by demonstrating the importance of uncomplicated psychotherapeutic approaches that are presented in a palatable format. It is possible that the addition of two interventions for separate disorders together can create complexity and uncertainty for the clinician so practical supervision regarding how to prioritise and integrate the content of treatment may be important (23–25). In fact, evidence from comorbidity training literature in related fields has consistently demonstrated the importance of supervision to the success of the implementation of integrated care initiatives (26–28).

Findings related to the *outer* and *inner setting* in this study corroborate previous findings in the AOD implementation literature about the importance clinicians place on patient needs and preferences when deciding whether or not to implement what might be considered to be a new intervention (21, 29, 30), along with findings about the importance of strong organisational learning climates that involve supportive training and supervision from directors and supervisors such as allocated time for learning (29, 31–33). The implementation of integrated care for comorbidity in mental health settings has also emphasised the importance of leadership engagement (34–36) and establishing a learning environment that allows for helpful reflection (28) with ongoing learning activities including consultation, supervision and case reviews (27). Interestingly, while resource allocation and inter-agency relationships were not distinguishing features of implementation effectiveness in this setting, they have been identified as primary barriers to the uptake of integrated care in mental health settings (27, 37–40).

Previous studies evaluating *characteristics of individuals* have identified barriers such as a lack of knowledge about evidenced-based approaches (22) or facilitators such as having more formal training (41, 42), positive attitudes toward (43, 44) or increased exposure to Aletraris et al. (45) evidence-based treatments, and an increased willingness to try new practises (29). In contrast, findings from the current study suggest that self-efficacy can be a powerful facilitator of the implementation. Another important departure from existing research is the finding that specific attributes of the individuals involved (such as feeling under-valued, feeling vulnerable, or having a particular practise habit), may present barriers to implementation and warrant further investigation.

Lastly, while there is limited existing research into the *process* domain (46), clinical champions have generally been perceived as a facilitator of implementation efforts (47–50). There is also evidence to suggest that clinical champions contribute to a faster uptake and sustained use of the intervention (51), and that they can assist with generating enthusiasm amongst staff, despite systemic barriers (48, 52, 53). Again, although various aspects of the process domain were found to have a neutral impact on the implementation in this context, formalising relationships (to ensure commitment and accountability) and documenting expectations and goals have been found to improve the uptake of integrated care in mental health agencies (27).

## Limitations

The main limitations of this study are its small sample size and non-randomised design, which limit the generalisability of findings. Although gaps in the CFIR have now been identified and more comprehensive frameworks exist (e.g., the Exploration, Preparation, Implementation, Sustainment framework) (54), it was considered to be the most appropriate framework at the time this study was designed. It is also important to note that this study represents one of few attempts worldwide to evaluate direct stakeholder accounts of implementation effectiveness for comorbidity care using a comprehensive implementation framework.

## Conclusion

In accordance with the barriers identified, future comorbidity training programs might ensure a positive learning environment within the organisation such as allocated time for learning. Our results also revealed that the implementation of the PCC package was facilitated by provision of a credible clinical supervisor, strong leadership engagement and an active clinical champion. The study has implications for services who manage comorbid substance use and mental disorders, a complex clinical problem often associated with poor treatment outcomes. Viewed alongside comparable implantation efforts in mental health settings, findings from this study suggest that the AOD setting may be less vulnerable to difficulties arising from disrupted networks and limited resources. Future research into barriers and facilitators specific to drug and alcohol clinicians might benefit from an exploration of other factors such as geographic location of services or a comparison of clinicians treating comorbidity in drug and alcohol vs. mental health service settings.

## Data Availability Statement

The raw data supporting the conclusions of this article will be made available by the authors, without undue reservation.

## Ethics Statement

The studies involving human participants were reviewed and approved by Human Ethics Review Committees of the Sydney Local Health District South Western Sydney Local Health District, Central Coast Local Health District Hunter New England Research Ethics and Governance Office which covered two participating services Mid North Coast Local Health District. The patients/participants provided their written informed consent to participate in this study.

## Author Contributions

KM, AB, PH, and MT contributed to study conception and design, supervision of the project, data analysis, and data interpretation of the main study. EL was the project coordinator and contributed to study coordination, clinician recruitment, data collection, and maintenance. GU, KW, and VG contributed to study coordination, data collection, data maintenance (cleaning and checking). VG contributed as Senior Clinical Psychologist and contributed to study implementation and data interpretation. SC and DR contributed as site investigators. EL conceptualised the paper, led the analysis, interpretation, and writing of manuscript. All authors approved the final manuscript.

## Funding

This study was supported by a Translational Research grant from the New South Wales Health (PH, KM, and AB), a Translational Research Fellowship (KM), a Research Training Program (EL), and MRFF/NHMRC Practitioner Research Fellowship (PH). The authors wish to thank our clinical colleagues who assisted with recruitment at the participating centres. The Comorbidity Guidelines was funded by the Australian Government Department of Health.

## Conflict of Interest

The authors declare that the research was conducted in the absence of any commercial or financial relationships that could be construed as a potential conflict of interest.

## Publisher's Note

All claims expressed in this article are solely those of the authors and do not necessarily represent those of their affiliated organizations, or those of the publisher, the editors and the reviewers. Any product that may be evaluated in this article, or claim that may be made by its manufacturer, is not guaranteed or endorsed by the publisher.
